# *N*-Butyrylated hyaluronic acid ameliorates gout and hyperuricemia in animal models

**DOI:** 10.1080/13880209.2019.1672755

**Published:** 2019-10-17

**Authors:** Lanzhou Li, Di Wang, Xueju Wang, Ruifeng Bai, Chunyu Wang, Yin Gao, Tassos Anastassiades

**Affiliations:** a Key Laboratory for Molecular Enzymology and Engineering of Ministry of Education, School of Life Sciences, Jilin University, Changchun, China;; b Pathology Department of China–Japan Union Hospital, Jilin University, Changchun, China;; c State Key Laboratory of Supramolecular Structure and Materials, Jilin University, Changchun, China;; d Division of Rheumatology, Department of Medicine, Queen’s University, Kingston, Canada

**Keywords:** Cytokines, ROS, *N*-acylation

## Abstract

**Context:** Hyaluronic acid (HA) plays critical roles in the structural skeleton, joint lubrication, renal function and cell signaling. We previously showed that partially *N*-butyrylated, low molecular weight, hyaluronic acid (BHA) exhibited an anti-inflammatory effect in cultured human macrophage, where inflammation was induced either by a TL-4 agonist or the low molecular weight HA itself, in dose-dependent fashion.

**Objectives:** To investigate the anti-inflammatory, antioxidative, and antihyperuricemic effects of BHA using animal models of acute gouty arthritis and hyperuricemia.

**Materials and methods:** The anti-inflammatory effect of articular BHA (10 and 50 μg) injections was evaluated by measuring joint swelling and the serum levels of inflammatory cytokines in a model of acute gouty arthritis induced by intra-articular injection of monosodium urate crystals in Wistar rats (*n* = 10/group), in comparison to the control group with saline injection. Antioxidative and antihyperuricemic activities were investigated using intraperitoneal injections of oteracil potassium and yeast extract hyperuricemic Balb/C mice, which were treated with intraperitoneal injection of BHA at day 6–8 in the model.

**Results:** In the gouty arthritis rat model, BHA at a higher dosage (50 μg) demonstrated a strong anti-inflammatory effect by reducing the degree of articular swelling and the serum levels of IL-1β, IL-8, IFN-γ, and MCP-1 by 5.56%, 6.55%, 15.58% and 33.18%. In the hyperuricemic mouse model, lower dosage BHA (10 μg) was sufficient to provide antioxidative activities by significantly decreasing the ROS levels in both serum and liver by 14.87% and 8.04%, while improving liver SOD by 12.77%. Intraperitoneal injection of BHA suppressed uric acid production through reducing liver XO activity by 19.78% and decreased the serum uric acid level in hyperuricemic mice by 30.41%.

**Conclusions**: This study demonstrated for the first time that BHA exhibits anti-inflammatory, antioxidative and antihyperuricemic effects *in vivo*, suggesting a potential therapeutic application of BHA in gouty arthritis and hyperuricemia.

## Introduction

Gout is one of the most prevalent metabolic disorders and is accompanied by a high level of uric acid in the blood and monosodium urate (MSU) crystal precipitation in joints and tissues. Joint swelling and pain of sudden onset are the major symptoms of acute gout (Perez-Ruiz et al. 2009). In the absence of treatment, an acute gout attack can reoccur, causing severe pain and stiffness due to progressive joint tissue and bone deterioration (Dalbeth et al. 2012; Roddy and Doherty 2012). Moreover, gout can lead to cytokine overproduction and in association with cardiovascular risk factors and drug side effects leading to organ failure remains a challenge in treatment and in maintaining quality of life (Busso and So 2010; Desai et al. 2017; Terkeltaub 2017).

A primary pathogenic mechanism of gouty arthritis is that MSU crystals activate monocytes cells after being endocytosed, thus promoting the secretion of inflammatory factors such as tumor necrosis factor-α (TNF-α) and interleukins-1β (IL-1β), which will then cause an influx of inflammatory cells and strengthen the inflammatory reaction (Petrilli and Martinon 2007). Inflammatory cytokines such as IL-1β and IL-8 are important factors in the pathogenesis of gouty arthritis, and thus inhibiting their production may constitute one strategy for managing difficult cases (So et al. 2007; Busso and So 2010; Kienhorst et al. 2015), although it is agreed that adequate therapy generally requires both urate lowering and anti-inflammatory approaches (Keenan and Schlesinger 2016). The clinical presentation of gout can be quite variable, and the role of innate immunity, and inflammation activation by metabolic processes has been reviewed (Busso and So 2010; Terkeltaub 2017).

It is generally agreed that hyperuricemia, which maybe asymptomatic, has a close relationship with gout (Campion et al. 1987; Nuki and Simkin 2006), and thus uric acid (UA) metabolism plays a critical role in the pathogenesis of acute and chronic gout. The up-regulation of xanthine oxidase (XO) which directly catalyzes the production of UA (Kanemitsu et al. 2017) and a deficiency of urate oxidase that is essential for UA metabolism can lead to hyperuricemia (Wu et al. 1989). Numerous reactive oxygen species (ROS) products are generated along with UA production (Meneshian and Bulkley 2002), which will promote oxidative stress, thus disrupting the biological redox equilibrium *in vivo* and ultimately damaging cellular functions (Valko et al. 2007).

Current pharmacological treatment of gout is mainly classified as drugs for treating acute attacks and for lowering UA levels (Harrold et al. 2009; Terkeltaub 2009). Drugs for the acute attacks include colchicine (COL) and nonsteroidal anti-inflammatory drugs (NSAIDs) and sometimes intra-articular or oral glucocorticoids (van Echteld et al. 2014; Dalbeth et al. 2016). Most NSAIDs act as nonselective inhibitors of cyclooxygenase to exert their anti-inflammatory and analgesic effects (Knights et al. 2010). However, the use of COL and NSAIDs may cause gastrointestinal discomfort, diarrhea, kidney problems and increase the risk of heart disease as well as cause other side-effects (Garcia Rodriguez and Hernandez-Diaz 2001; Knights et al. 2010; van Echteld et al. 2014).

The UA-lowering drugs that are used to prevent of attacks of gout include xanthine oxidase inhibitors, URAT1 inhibitors, and uricosuric drugs. The xanthine oxidase inhibitors (such as febuxostat) and URAT1 inhibitors (such as lesinurad, pegloticase and allopurinol, AL) inhibit UA production (Jordan et al. 2007), while uricosuric drugs (such as probenecid) (Mason 1954) increase the excretion of uric acid in urine. However serious side-effects limit the use of these drugs (Rizzuto et al. 1965; Bohm et al. 2016; Chung et al. 2016; Guttmann et al. 2017; Sanchez-Nino et al. 2017) and therefore, the development of a comprehensive and safe alternative medication for managing both the acute attack of gout as well as the hyperuricemia is still needed. Further, a single compound with anti-inflammatory activity for the acute attacks of gout as well as lowering elevated urate levels would be advantageous.

**Figure 1.**
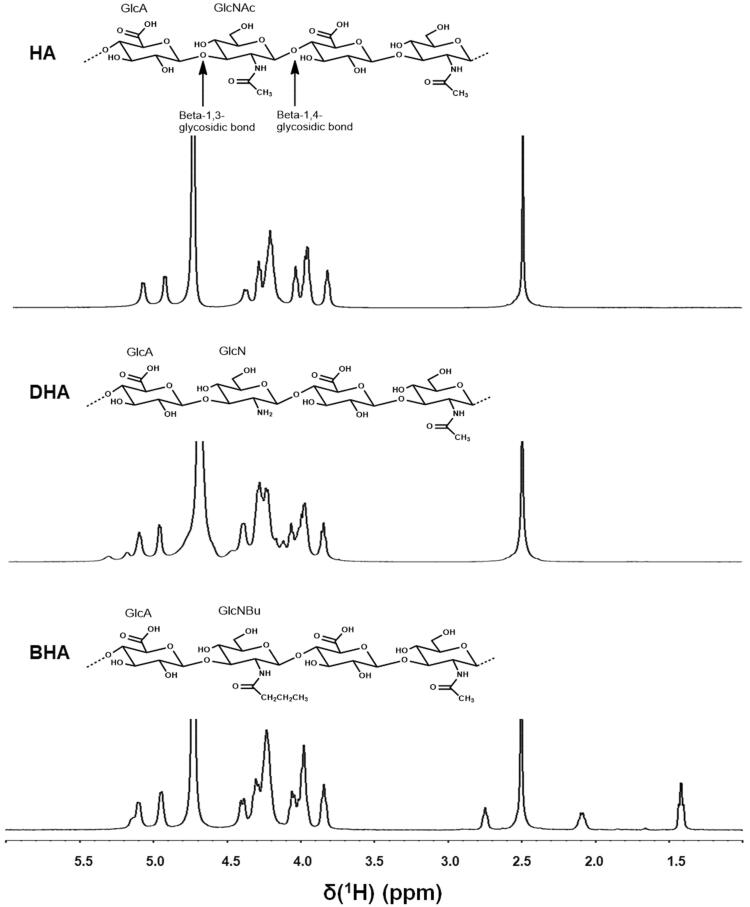
1 H NMR spectra of HA, DHA and BHA. Polysaccharides were prepared at a concentration of 15 mg mL– in D_2_O. 1H NMR spectra of HA, DHA and BHA were recorded at 348 K with a 500 MHz spectrometer. HA: hyaluronic acid; DHA: partially deacetylated HA; BHA: partially butylated HA; GlcA: d-glucuronic acid; GlcNAc: *N*-acetyl-d-glucosamine; GlcN: d-glucosamine; GlcNBu: *N*-butyl-d-glucosamine.

Hyaluronic acid (HA) is a linear polysaccharide composed of simple repeating disaccharide units of *N*-acetyl-d-glucosamine (GlcNAc) and d-glucuronic acid (GlcA) (Figure 1) is the main component of the extracellular matrix and is a key component of articular cartilage and is abundant in the synovial fluid (Dahl et al. 1985). The intra-articular injection of high molecular weight HA (HMW HA) has shown moderate improvement in pain in models of arthritis including gout (Marcotti et al. 2018). HMW HA and its cross-linked preparations have been used clinically in equine and human osteoarthritis of weight-bearing joints (Bannuru et al. 2014). HA is also an important component of the renal interstitial tissue, where it is considered to regulate kidney function during normal and pathological conditions (Stridh et al. 2012).

We had previously shown (Babasola et al. 2014) that the *N*-butyrylated HA (BHA) with reduced molecular weight significantly inhibited the production of proinflammatory cytokines, such as the TNF-α, IL-1, IL-6 and IL-8 in the lipopolysaccharide (LPS) stimulated human cultured macrophages, while other *N*-acyl substitutions were less effective. In this publication we studied the pro-inflammatory effects of the semisynthetic polymers over a wide range of concentrations. We demonstrated induction of proinflammatory cytokine production by the low molecular weight HA. This HA polymer was initially deacetylated and then fully reacetylated, to an identical extent as the BHA, so that it could be precisely compared to BHA. In addition, we showed that increasing concentrations of the TL4 agonist, lipopolysaccharide from *E. coli*, in the presence of a fixed amount of BHA resulted in much lower of proinflammatory cytokine production from the macrophages than in its absence.

In the current study, we examine the anti-inflammatory effect and alleviation of joint swelling provided by BHA in rats where experimental gout was induced by the intra-articular injection of MSU crystal. The antihyperuricemic effects of BHA were also explored in mice where hyperuricemia was induced by oteracil potassium (OXO) and yeast extract powder. The underlying mechanisms related to oxidative stress and inflammation were also investigated.

## Materials and methods

### Synthesis and characterization of BHA

The BHA was synthesized by two step reaction as previously described (Babasola et al. 2014) and purified by 5 days dialysis against deionized water. Water was changed twice a day.

### ^1^H NMR analysis of DHA and BHA

To characterize the structure of DHA and BHA, ^1^H NMR spectra of 10 mg samples were recorded in D_2_O at 348 K using a 500 MHz Bruker nuclear magnetic resonance (NMR) spectrometer The degree of deacetylation can be calculated according to the equation: Deacetylation (%) = (1.0−(Y/1.5)100) (Babasola et al. 2014). The spectrum of BHA in Figure 1 exhibited additional –CH_2_CH_2_CH_3_ proton signals, the butylation-to-acetylation ratio of the sample of BHA was the integration ratio of methyl protons in the –CH_2_CH_2_CH_3_ moiety to the total methyl protons in the GlcNAc and –CH_2_CH_2_CH_3_ moieties.

### Molecular weight estimation of HA, DHA and BHA

**Figure 2.**
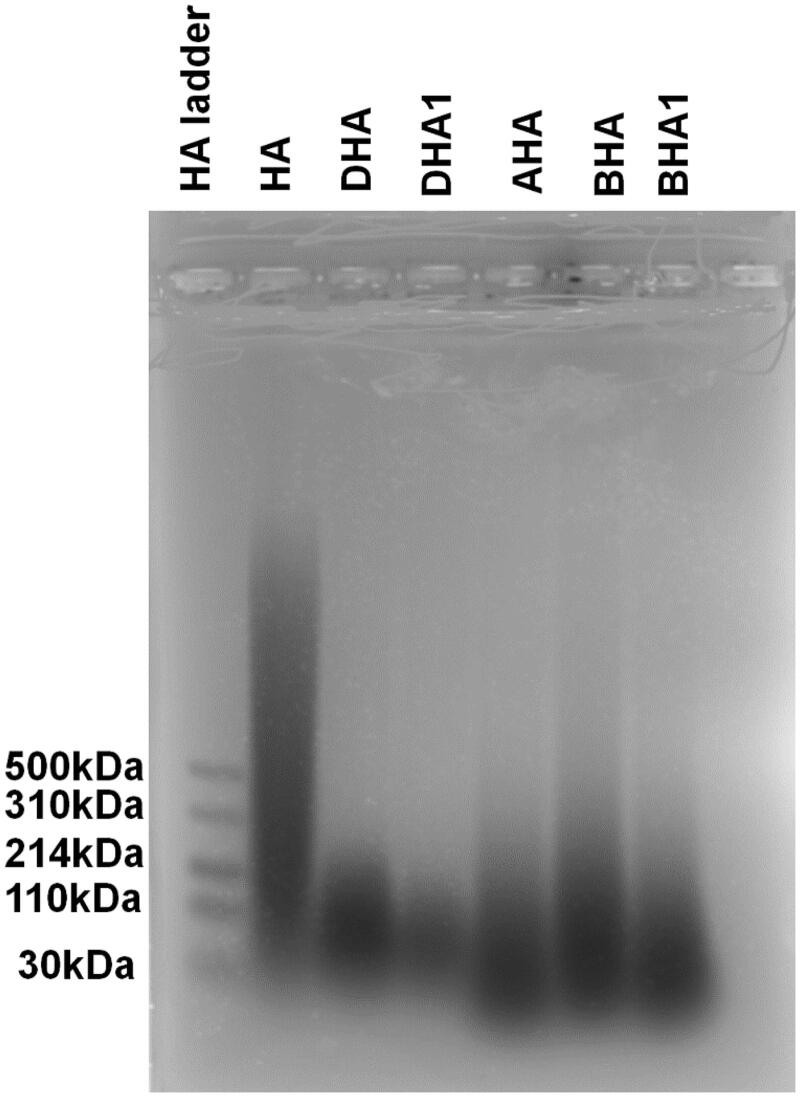
Electrophoretic separation of HA and its derivatives. The HA ladder (standard) has a range of molecular weight of HA from 500 to 30 kDa. The amounts of analytes that were loaded on the gel are as follows: 7.5 μg of native HA; 7.5 μg of DHA; 5 μg of DHA1, prepared via the same method as to prepare DHA but from a different batch; 7.5 μg of AHA, partially deacetylated HA and then reacylated with acetic anhydride; 7.5 µg of BHA; 7.5 μg of BHA1, prepared via the same method as to prepare BHA, but from a different batch.

The molecular weights of DHA and BHA were estimated via electrophoresis as previously described (Babasola et al. 2014). Loading samples were prepared as 15 μL solutions with 3 μL of loading buffer plus different amount of the analytes indicated in Figure 2 (HA, DHA, BHA). This staining procedure was performed in the dark over a duration of 48 h. For destaining, the gel was placed in 10% (v/v) ethanol, kept in the dark, destained for 48 h, during which time the destaining solution was replaced three times.

### Mass spectrometry analysis of HA, DHA and BHA

MS analyses of the samples were performed using a Triple-TOF 5600 mass spectrometer (SCIEX, Concord, Canada) equipped with an electrospray ionization source operated in the negative scanning mode. MS parameters, which were optimized by a 10 μg mL^−1^ HA solution via a syringe pump, were as follows: Source temperature = 550 °C, ion spray voltage = −4500 V, nebulizer gas (N2) pressure = 25 psi, heater gas (N2) pressure = 50 psi, Curtain gas pressure = 25 psi, DP = −100 V and CE = −35 eV. Samples at concentrations of 10 μg mL^−1^ were injected into the mass spectrometer via a syringe pump to scan for specific fragments corresponding to HA and its derivatives as shown in Table 1 via the TOF-MS scanning mode. Data acquisition was controlled by Analyst 1.6.1 software.

**Table 1.** The theoretical and observed molecular ion species in HA and HA derivatives along with their relative intensities.

			Observed *m*/*z*		
Molecular ions	Charge	Theoretical *m*/*z*	HA	DHA	BHA
Disaccharide of GlcNAc and GlcA	−1	396.1142	396.1160 (100%)	396.1138 (100%)	396.1139 (100%)
Tetrasaccharide of GlcNAc and GlcA	−1	775.2257	775.2299 (20.0%)	775.2268 (23.3%)	775.2309 (15.7%)
Tetrasaccharide of GlcNAc and GlcA	−1	797.2076	797.2069 (13.7%)	797.2144 (5.5%)	797.2206 (10.8%)
Tetrasaccharide of GlcNAc and GlcA	−2	387.1089	387.1100 (8.0%)	387.1078 (12.0%)	387.1107 (7.2%)
Disaccharide of GlcN and GlcA	−1	354.1036		354.1053 (23.3%)	
Disaccharide of GlcNBu and GlcA	−1	424.1455			424.1462 (27.0%)

### Animal Experiments – The MSU crystal-induced acute gout model in the rat

Male Wistar rats (*n* = 50, 8 weeks: 160–200 g) were purchased from Liaoning Changsheng Biotechnology Company, Jilin, China (SCXK (Liao)-2015-0001). These rats were housed in plastic cages and maintained on a 12 h light/dark cycle (lights on 7:00–19:00 h) under standard laboratory conditions of 55% relative humidity and at 23 °C ± 1 °C. They were given standard chow (Liaoning Changsheng Biotechnology Company, Jilin, China) and tap water *ad libitum*. The protocols of the animal experiments were approved by the Animal Ethics Committee of Jilin University (Reference No. 201605).

### Protocol for inducing acute gout in rats by MSU crystal injection and treatment by BHA

**Figure 3.**
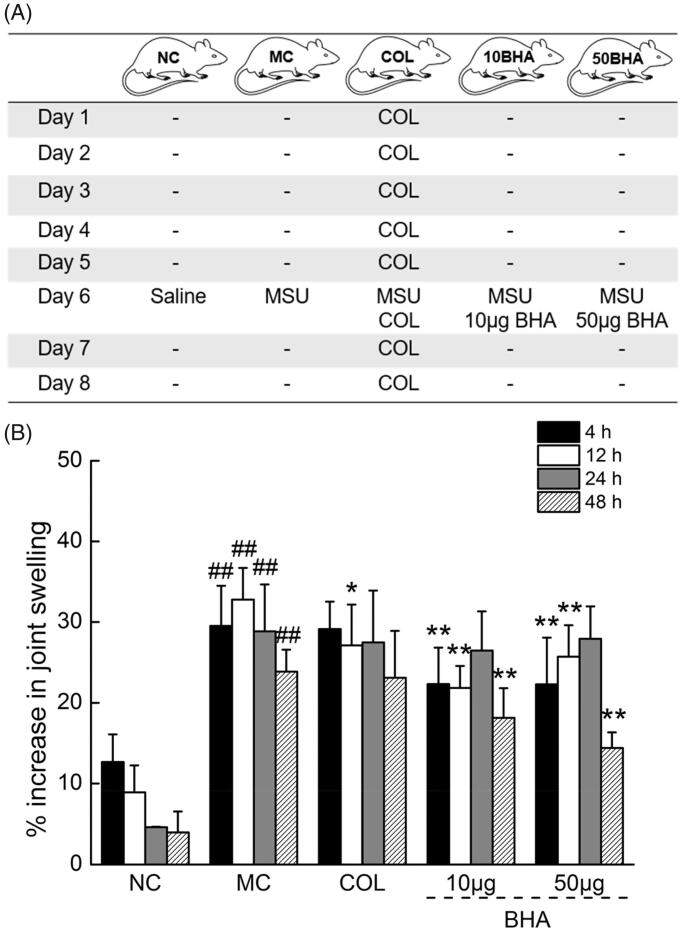
(A) Summary of the protocol for MSU crystal injection and drug administration in rats. COL (0.3 mg/kg/day) was orally administrated; MSU solution (100 μL, 30 mg mL^−1^) or MSU and BHA (10 or 50 μg) were intra-articular injected at the right ankles. (B) The effects of COL and BHA on the ankle swelling in MSU crystal-injected rats. The % decrease in swelling with time in the normal control group (NC) may be due to the physical damage caused by the needle injection of 0.9% sterile saline. Data are expressed as mean ± S.D. (*n* = 10) and were analyzed via a one-way ANOVA test followed by *post hoc* Dunn’s multiple comparison tests. ##*p* < 0.01 versus control rats, *p < 0.05 and **p < 0.01 versus model rats.

An experimental model of MSU-induced gouty arthritis established as described in the literature (Zhou et al. 2017) with modifications was used to evaluate the anti-inflammatory activity of BHA *in vivo* (Babasola et al. 2014). Rats were randomly divided into five groups (*n* = 10) as shown in Figure 3(A): (1) a control group (NC), (2) an acute gout model group (MC), (3) a 10 μg BHA administration group, (4) a 50 μg BHA administration group, (5) COL administration group, COL was orally administration for 8 days, 0.3 mg/kg/day. At day 6, the 100 μL of 30 mg mL^−1^ MSU solution was intra-articular injected at the right ankles of all rats except the NC group which injected with saline solution. In the 10BHA and 50BHA groups, intra-articular injections of 10 or 50 μg BHA along with MSU solution. About 1 μg or 5 μg BHA was dissolved into 10 mL of 30 mg mL^−1^ MSU solution, and 100 μL of 30 mg mL^−1^ MSU solution contains 10 or 50 μg BHA were administered according to the stated BHA doses. On the 8th day, 1 h after the administration of the final COL solution, blood was sampled from the caudal veins of the rats. Immediately after sampling the right ankle joints of the rats of all of the groups of the rats were collected and fixed in 4% paraformaldehyde. Serum was separated and stored at −80 °C until analysis.

### Measurement of joint circumferences in the acute gout rat model

The right ankle circumference of all groups of the rats at the certain time (4, 12, 24, 48 h) after the intra-articular injection step was measured with a line method (Huang et al. 2012). Briefly, a thin line without elasticity was used to determine the ankle circumference at the same position of each rat. Then the marked length of the line was read with a digital caliper (Shanghai Jianghua Tools). The increase in joint swelling was used to evaluate the gouty arthritis and calculated according to the % increase of the circumference compared to circumference prior to injection via the formula: The % increase in joint swelling = (*C_t_* − *C*_0_)/*C*_0_ × 100. In particular, *C*_t_ represents the circumference at time (*t*) (in h) and *C*_0_ represented the circumference at 0 h.

### Histopathological assessment of ankle joints in the acute gout rat model

The right ankles of mice were excised and fixed in 4% paraformaldehyde, and subsequently decalcified using 10% ethylenediaminetetraacetic acid. They were then dehydrated via processing in alcohol/xylene mixtures with different proportions and concentrations. The histological sections were stained with hematoxylin and eosin and assessed under a microscope (40× to 400×, final magnification). The histopathological changes of the joint synovium were assessed for the degree of inflammatory cell infiltrate, by an experienced histopathologist.

### Biochemical assays in the acute gout rat model

The serum levels of interleukin-1α (IL-1α), IL-1β, interleukin-6 (IL-6), interleukin-8 (IL-8), IL-10, interleukin-16 (IL-16), C-X-C motif chemokine 10 (CXCL10), monocyte chemoattractant protein 1 (MCP-1), macrophage inflammatory protein 1α (MIP-1α), interferon gamma (IFN-γ), TNF-α, tumor necrosis factor-β (TNF-β), nuclear factor κ-light-chain-enhancer of activated B cells (NF-κB), 6-keto-prostaglandin F-1α (6-Keto-PGF1α) and prostaglandin E2 (PGE2) in rats were determined by the ELISA method using related Elisa Kits (Yuanye Bio-Technology, Shanghai, China) according to the manufacturer’s instructions. The absorbance recorded in different concentrations that used to establish the calibration curves were shown in the Supporting Information Table S1.

### Animal experiments – the mouse hyperuricemia model

Male Balb/C mice (*n* = 50, 8 weeks: 18–22 g) were purchased from Yisi Experimental Animal Technology Company, Jilin, China (SCXK (Ji)-2016-0003). These mice were housed in plastic cages and maintained on a 12 h light/dark cycle (lights on 7:00–19:00 h) under standard laboratory conditions of 55% relative humidity and 23 °C ± 1 °C. They were given standard feed and tap water *ad libitum*. The protocols of the animal experiments were approved by the Animal Ethics Committee of Jilin University (Reference No. 201605).

### Protocol for inducing hyperuricemia in mice and treatment by BHA

**Figure 4.**
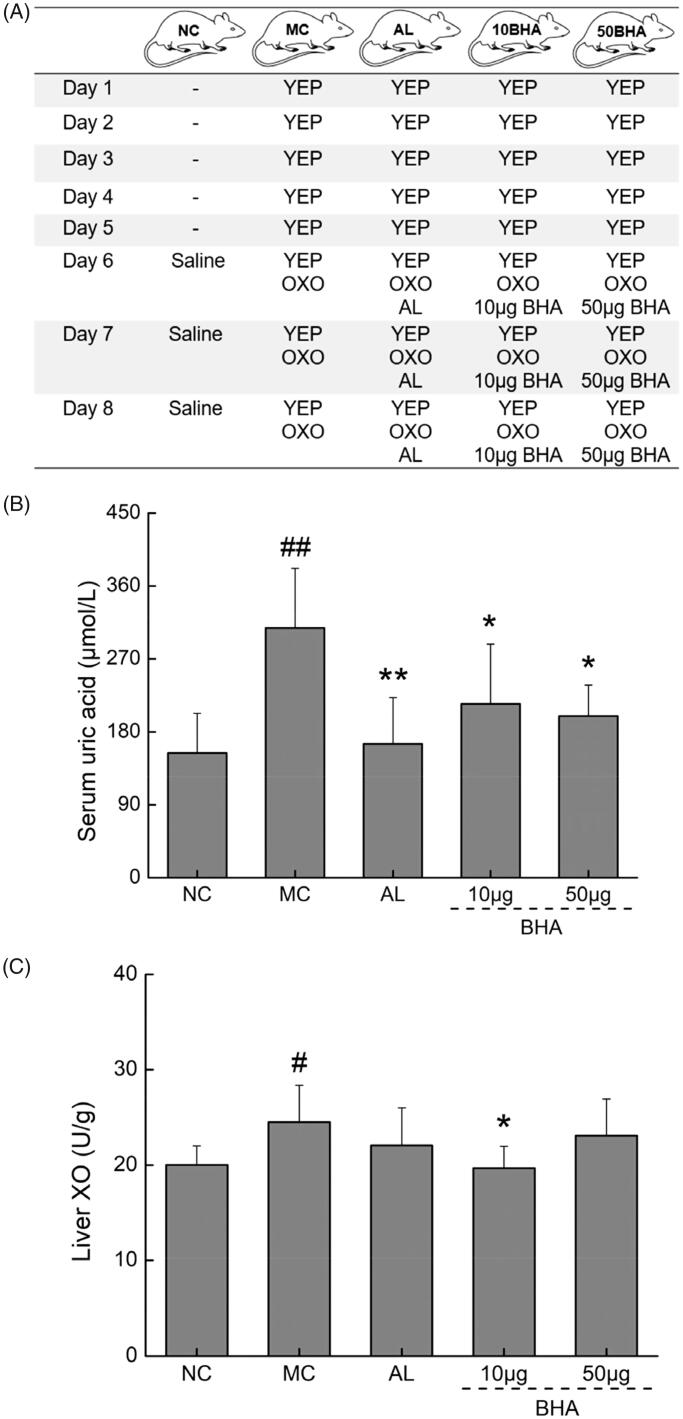
(A) Summary of the protocol for hyperuricemic mice. Yeast extract powder (YEP) (20 g kg^−1^) was orally administrated; AL (20 mg kg^−1^) was orally administrated; OXO (300 mg kg^−1^) or OXO and BHA (10 or 50 μg) were intraperitoneally administrated. (B) The effects of AL and BHA on the serum levels of UA in hyperuricemic mice. (C) The effects of AL and BHA on the XO levels in the liver. Data are expressed as mean ± S.D. (*n* = 10) and were analyzed via a one-way ANOVA test followed by *post hoc* Dunn’s multiple comparison tests. #*p* < 0.05 and ##*p* < 0.01 versus normal control, *p < 0.05 and **p < 0.01 versus model control.

A hyperuricemic mouse model was established by using uricase inhibitor and large amounts of purine via a method described in the literature with some modifications (Amat et al. 2015). Mice were randomly divided into five groups (*n* = 10) as shown in Figure 4(A): (1) the control group (NC), (2) hyperuricemic model group (MC), (3) AL administration group, (4) 10 μg BHA administrated group and (5) 50 μg BHA administration group. All groups of mice, except the NC group, were fed 20 g kg^−1^ yeast extract powder (YEP) by gavage for 8 days. From day 6 to 8, 300 mg kg^−1^ of OXO was intraperitoneally injected to all the mice except the control group, which injected with saline solution. In MC group, 20 mg kg^−1^ AL was orally administrated after 1 h of the OXO injection. In the 10BHA and 50BHA groups, intraperitoneal injections of 10 or 50 μg BHA along with OXO were conducted according to the required BHA doses from day 6 to 8 (1 mg or 5 mg BHA was dissolved into 20 mL saline solution, and 200 μL of saline solution contains 10 or 50 μg BHA were administered). At 1 h after the final drug administration, blood was sampled from the caudal veins of the mice, serum was separated, and their livers were quickly collected (Figure 4(A)). All samples were stored at −80 °C until assay measurements were performed.

### Measurement of the levels of UA and XO in hyperuricemia mouse models

The serum UA concentrations and XO levels in the serum and liver were determined with standard diagnostic kits (MAK077, MAK078, Sigma, USA) according to the manufacturer’s instructions. Tissue homogenate samples were prepared with a 0.9% sterile saline solution.

### Measurement of factors related to oxidative stress and renal function in hyperuricemic mice

The levels of reactive oxygen species (ROS, 43355), malondialdehyde (MDA) glutathione peroxidase (GSH-Px), superoxide dismutase (SOD), catalase (CAT), creatinine (Cr) and urea nitrogen in the serum and livers of mice were determined using ELISA Kits (Yuanye Bio-Technology, Shanghai, China) according to the manufacturer’s instructions.

### Proteome profiling on inflammatory factors of human plasma

Blood was sampled from six healthy volunteers and six patients suffering with acute gout attack enrolled in the First Hospital of Jilin University, Changchun, China. The clinical trial protocol was approved by the Ethics Committee of Jilin University (Reference NO. 2015-015). We adhered to the Guidelines on Humane Treatment to Lab Animals (published in 2009). Inflammatory factors of human plasma were assayed using a Proteome Profiler Human Cytokine Array Kit (Ary005b, R&D Systems Bio-Technology China, Minneapolis, MN), according to manufacturer’s instructions.

### Statistics

All results collected were expressed as mean ± S.D. One-way analysis of variance (ANOVA) was performed to evaluate statistical significance of the data, and this was followed by *post hoc* Dunn’s multiple comparisons test by SPSS 19.0 Software (IBM corporation, Armonk, USA). *p* values < 0.05 were considered to be statistically significant.

## Results

### ^1^H NMR analysis of HA, DHA and BHA

The NMR data are as follows, HA: *δ*2.50 (s, –CH_3_), *δ*4.40–3.83 (m, other Hs on carbohydrate rings), *δ*5.09–5.08 (d, anomeric H on GlcNAc of HA), *δ*4.94–4.93 (d, anomeric H on GlcA of HA). DHA: *δ*2.50 (s, –CH_3_), *δ*4.45–3.84 (m, other Hs on carbohydrate rings), *δ*5.10 (d, anomeric H on GlcNAc of HA), *δ*4.95 (d, anomeric H on GlcA of HA), *δ*5.31 (d, anomeric H on GlcNAc of DHA), *δ*5.18 (d, anomeric H on GlcA of DHA). BHA: *δ*2.50 (s, –CH_3_ of GlcNAc), *δ*4.40–3.83 (m, other Hs on carbohydrate rings), *δ*5.10–5.09 (d, anomeric H on GlcNAc of HA), *δ*4.94–4.93 (*d*, anomeric H on GlcA of HA), *δ*5.13 (d, anomeric H on GlcNAc of BHA), *δ*5.10–5.09 (d, anomeric H on GlcA of BHA), *δ*2.74 (t, –CH_2_ of GlcNBu of BHA), *δ*2.09–2.08 (m, –CH_2_ of GlcNBu of BHA), *δ*1.42 (*t*, –CH_3_ of GlcNBu of BHA). As shown in Figure 1, the GlcNAc-GlcA disaccharide units of HA underwent partial deacetylation, and some of the GlcNAc was converted to GlcN to yield DHA. The anomeric proton corresponding to GlcNAc in the GlcNAc-GlcA unit was observed at 5.09–5.08 ppm as a doublet. The anomeric proton of GlcA in the GlcNAc-GlcA unit was also observed as a doublet at 4.94–4.93 ppm. The newly visible smaller peaks at 5.18–5.31 ppm corresponded to the anomeric protons of the GlcN-GlcA unit. The anomeric proton of GlcN in the GlcN-GlcA unit was observed at 5.09–5.08 ppm as a doublet. The anomeric proton of GlcA in GlcN-GlcA unit was also observed as a doublet at 4.94–4.93 ppm. In the spectrum of DHA, the integration ratio of the three methyl protons to the anomeric protons was calculated to be 1.13. From this ratio, the percentage of deacetylation was calculated to be 24.8%. The spectrum of BHA shows additional –CH_2_CH_2_CH_3_ proton signals indicating that a reacylation reaction had occurred and butylation-to-acylation ratio was calculated to be 25.4%.

### Molecular weights of HA, DHA and BHA

The molecular weights of HA and its derivatives were estimated by agarose gel electrophoresis, as shown in Figure 2. The low molecular weight HA ladder has a molecular mass range of 500 kDa to 30 kDa and was used to estimate the molecular weights of HA, DHA and BHA. The purchased HA had a range of molecular masses from 1800 kDa to 30 kDa, while DHA and DHA1 had a molecular weight of ∼60 kDa. The samples of DHA and DHA1 were prepared by the same methods but from different batches, indicating that the preparation of 60 kDa DHA via the hydrazinolysis reaction was reproducible. The molecular weight of BHA and BHA1 was estimated to be ∼30 kDa. These samples were prepared via the same methods but from the different batches, indicating that the reacylation reaction was also reproducible. AHA denoted the partially deacetylated HA that was reacylated with acetic anhydride. The molecular weight of AHA was also estimated to be ∼30 kDa, suggesting that the reacylation method for preparing different HA-derivatives yielded products with similar molecular weights.

### Mass spectrometry analysis of HA, DHA and BHA

Following injection into the Q-TOF MS system via a syringe pump, the sample solutions were scanned in the TOF-MS mode. The observed *m/z* value was very similar to the predicted *m*/*z* value, as shown in Table 1. Singly charged disaccharides of GlcNAc and GlcA (*m*/*z* 396.1160), as well as singly (*m*/*z* 775.2257, 797.2076) and doubly (*m*/*z* 387.1089) charged tetrasaccharides of GlcNAc and GlcA were observed in the mass spectra of the HA sample. The presence of additional singly charged disaccharide of GlcN and GlcA (*m*/*z* 354.1053) as observed via TOF-MS spectra showed that the sample of DHA was composed of partially deacetylated HA. The additional singly charged disaccharide of GlcNBu and GlcA (*m*/*z* 424.1462) observed in the TOF-MS spectra of BHA revealed that the sample contained partially butylated HA. In these specific fragments, singly charged disaccharides of GlcNAc and GlcA (theoretical *m*/*z* 396.1142) were the most abundant species in all of the samples, and thus we used this signal as the target peak. The relative intensities of the other relevant peaks were calculated based on the target peak as shown in Table 1.

### Effects of BHA on rats with MSU-induced acute gout

As shown in Figure 1(A), MSU, either alone or mixed with BHA (at two doses) are administered intra-articularly to the right ankle on day 6 (see Materials and methods section). The MSU injection alone significantly increased the % swelling of the right ankles in rats (by 29.5%, 32.8%, 28.8% and 23.9% at 4, 12, 24 and 48 h after MSU injection, respectively, *p* < 0.01, *F* = 66.51 to 203.12) compared with control rats (Figure 3(B)). Treatment with 0.3 mg kg^−1^ COL significantly suppressed ankle swelling at 12 h (*p* < 0.05, *F* = 6.307) in comparison with MC group, but the effects did not reach statistical significance (*p* < 0.05, *F* = 0.04 to 0.22) at 4, 24 and 48 h. BHA injected along with MSU at the doses of 10 and 50 μg suppressed the ankle swelling of each rat at 4, 12 and 48 h (*p* < 0.01, *F* = 9.06 to 68.75) in comparison with the MC group. The treatment with 10 µg of BHA provided the best suppression of swelling at 12 h, and the swelling ratio was reduced by 11.0% (*p* < 0.01, *F* = 42.11). Additionally, treatment with 50 μg of BHA provided the best swelling suppression at 48 h, with a swelling ratio reduction of 9.5% (*p* < 0.01, *F* = 68.75).

**Figure 5.**
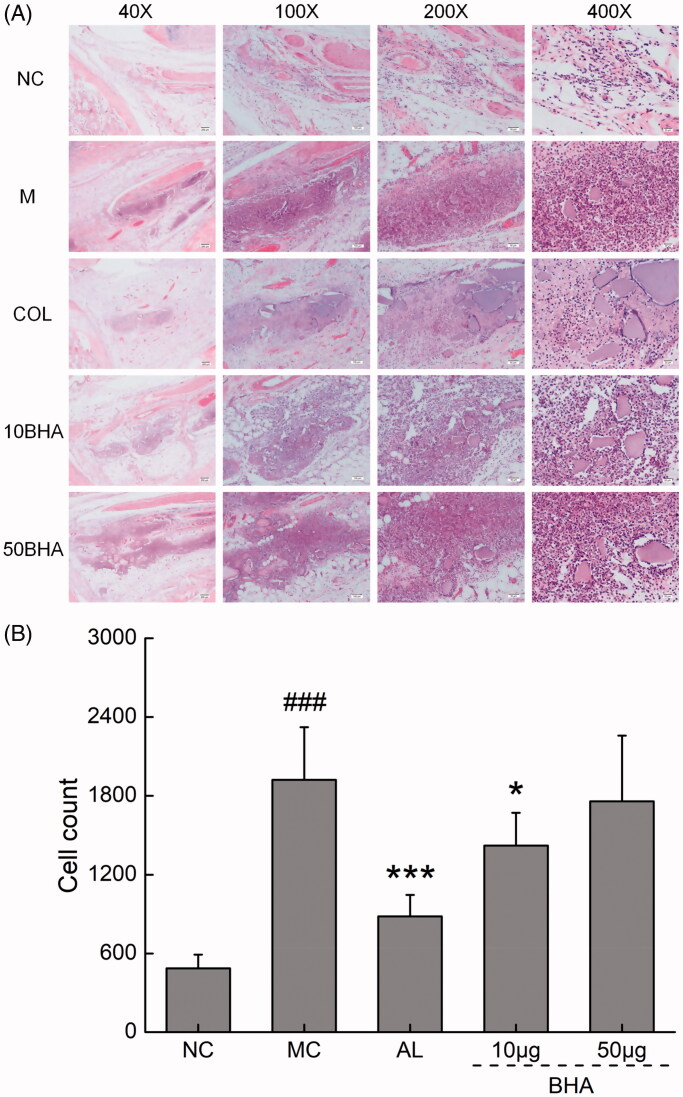
(A) Histopathological assessment of synovium in ankle joints of rats after H&E staining. The right ankles of mice were excised and fixed in 4% paraformaldehyde, and subsequently decalcified using 10% ethylenediaminetetraacetic acid. They were then dehydrated via processing in alcohol/xylene mixtures with different proportions and concentrations. The histological sections were stained with hematoxylin and eosin. The histopathological changes of the joint synovium were assessed for the degree of inflammatory cell infiltrate, by an experienced histopathologist. Microscopy at magnifications of 40×, 100×, 200× and 400× is shown for typical areas for each of the five groups. Normal rats (NC), displayed normal synovium. Increased inflammatory cell infiltration was noted in the synovium of MSU crystal-injected rats (MC). Treatment with COL, and 10 µg of BHA partially prevented the pathological changes seen in the MSU crystal-injected rats. (B) Quantification of the pro-inflammatory cells. Data are expressed as mean ± S.D. (*n* = 5), ###*p* < 0.001 relative to NC group (n = 5); *p < 0.05 and ***p < 0.001 relative to MC group.

Histopathologically, the injection of MSU crystals caused pronounced inflammatory cell infiltration in the synovium compared to the normal controls, as shown in Figure 5(A). The treatment with COL and the low dose of BHA attenuated the inflammation reaction in terms of that fewer inflammatory cells were observed in the groups of COL and 10BHA. As seen in Figure 5(B), the number of inflammatory cell infiltration was significantly elevated in the group with acute gout attack (MC) compared to the control group. The COL and 10BHA treatments significantly reduced infiltration of proinflammatory cells, while the higher dose BHA (50BHA) was not as effective as the 10BHA dose (Figure 5(B)), suggesting lower dosage of BHA provide a better effect in attenuating inflammatory cell inflation.

As inflammatory factors are an important basis for gout attack (Petrilli and Martinon 2007; Terkeltaub 2017), in the present study, we have detected and identified 15 inflammatory factors (Table 2). The data used to establish the calibration curves for the calculation were listed in the Supporting Information Table S1. In comparison to the control group, the contents of MCP-1 and 6-Keto-PGF1α were significantly increased (*p* < 0.05, *F* = 5.25–14.97), IL-10 had decreased dramatically (*p* < 0.05, *F* = 5.27), while IFN-γ and NF-κB were not significantly improved (*p* > 0.05, *F* = 1.08 to 4.52) in the serum of rats that had received MSU injections. Compared to rats injected with MSU, oral administration with 0.3 mg kg^−1^ COL only decreased the level of MCP-1 by 9.93% (*p* < 0.05, *F* = 7.60) in the serum. Intra-articular treatment with 10 μg BHA significantly decreased the level of IL-8 and MCP-1 by 7.13% and 7.76% (*p* < 0.05, *F* = 5.17 to 8.72), respectively, and increased IL-10 by 9.49% (*p* < 0.05, *F* = 5.875), indicating that the effect of BHA is more obvious in such lower dosage. Lastly, treatment with 50 μg BHA significantly decreased the level of IL-1β, IL-8, MCP-1 and IFN-γ by 5.56%, 6.55%, 15.58% and 33.18% (*p* < 0.05, *F* = 5.84–14.29), respectively.

**Table 2.** The effects of COL and BHA on the pro-inflammatory cytokines in rats with MSU-induced acute gout.

	NC	MC	COL	10BHA	50BHA
IL-1α (pg/mL)	79.4 ± 5.4	72.2 ± 7.0	74.3 ± 12.6	71.5 ± 11.2	77.4 ± 13.0
IL-1β (pg/mL)	19.6 ± 0.5	19.9 ± 0.5	19.1 ± 1.2	19.6 ± 1.6	18.8 ± 0.8*
IL-6 (pg/mL)	71.1 ± 4.7	74.2 ± 6.2	74.7 ± 7.9	78.5 ± 5.6	75.0 ± 5.3
IL-8 (pg/mL)	232 ± 10.7	231.8 ± 8.6	222.7 ± 11.5	213.8 ± 11.4*	216.6 ± 11.6*
IL-10 (pg/mL)	11.8 ± 0.6	11.0 ± 0.8#	11.5 ± 0.7	12.0 ± 0.9*	11.6 ± 1.1
IL-16 (pg/mL)	397.9 ± 31.1	405.7 ± 20.5	416.7 ± 16.1	399.3 ± 14.8	401.2 ± 25.1
IP-10 (pg/mL)	143.8 ± 7.7	158.4 ± 23.4	153.5 ± 20.3	154.3 ± 18.0	150.5 ± 7.5
MCP-1 (pg/mL)	320.2 ± 16.0	345.8 ± 24.2#	311.5 ± 20.8*	321.2 ± 20.5*	292.0 ± 27.8**
MIP-1α (pg/mL)	289.8 ± 25.1	289.2 ± 20.3	294.6 ± 21.5	282.8 ± 23.6	283.9 ± 12.7
IFN-γ (pg/mL)	99.6 ± 26.0	112.8 ± 21.6	88.2 ± 35.0	107.4 ± 7.6	75.4 ± 31.7*
TNF-α (pg/mL)	154.6 ± 13.4	156.2 ± 13.0	154.8 ± 8.5	155.9 ± 11.4	152.7 ± 12.1
TNF-β (pg/mL)	121.4 ± 28.2	127.7 ± 22.8	124.8 ± 10.5	139.1 ± 25.7	129.0 ± 21.9
NF-κB (pg/mL)	358.1 ± 97.0	501.3 ± 102.9	494.6 ± 132.2	589.8 ± 104.8	513.0 ± 74.1
6-Keto-PGF1α (pg/mL)	214.5 ± 17.5	251.3 ± 21.7#	266.3 ± 12.7	257 ± 20.8	262.0 ± 10.8
PGE2 (pg/mL)	195 ± 10.5	199.5 ± 8.6	207.7 ± 10.0	209.7 ± 17.3	193.2 ± 12.7

Data are expressed as mean ± S.D. (*n* = 10) and analyzed via a one-way ANOVA test followed by post-hoc Dunn’s multiple comparison tests.

# *p* < 0.05 versus control rats.

*p < 0.05 and **p < 0.01 versus model rats.

### Effects of BHA on hyperuricemic mice

A reduction in elevated levels of uric acid needs to be established to demonstrate effective antihyperuricemic agents (Liu et al. 2008). In comparison with the normal mice, the levels of serum uric acid (UA) was enhanced significantly (*p* < 0.01, *F* = 19.99) establishing the presence of hyperuracemia (Figure 4(B)). Intra-pertiotneal administration of BHA significantly suppressed serum UA levels (*p* < 0.05, *F* = 5.26–7.23). Oral administration with 20 mg kg^−1^ AL suppressed the serum UA level by 46.38% (*p* < 0.01, *F* = 11.65).

XO plays an important role in the production of UA and the treatment of hyperuricemia (Wu et al. 1989; Kanemitsu et al. 2017). In our hyperuricemic mice, liver XO levels were intensified by 22.52% (*p* < 0.05, *F* = 7.36; Figure 4(C)) in comparison to those observed in mice belonging to the control group. Compared to untreated mice, the administration of 10 μg BHA reduced XO levels in the liver by 19.78% (*p* < 0.05, *F* = 7.80; Figure 4(C)).

**Figure 6.**
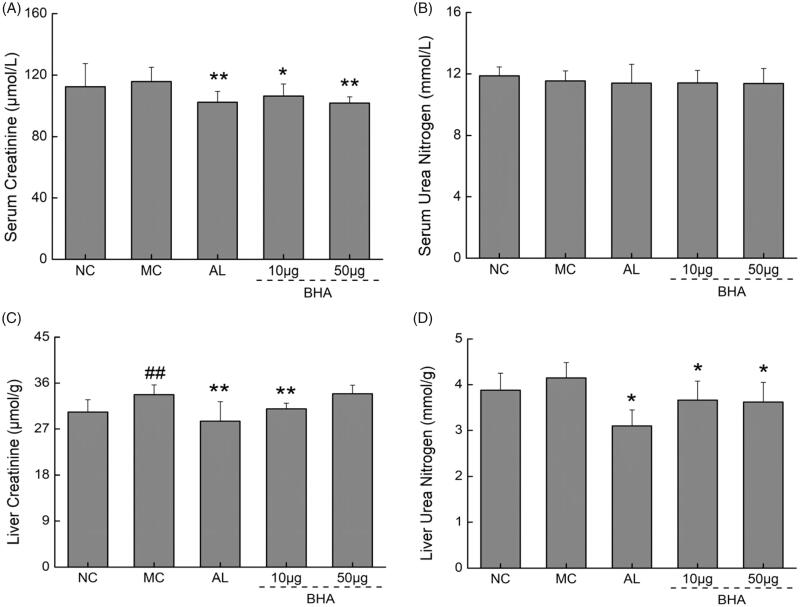
(A) The effects of AL and BHA on the serum level of Cr in hyperuricemic mice. (B) The effects of AL and BHA on the serum level of urea nitrogen in hyperuricemic mice. Data are expressed as mean ± S.D. (*n* = 10) and were analyzed via a one-way ANOVA test followed by *post hoc* Dunn’s multiple comparison tests. ##*p* < 0.01 versus normal control, *p < 0.05 and **p < 0.01 versus model control.

XO catalyzes the production of ROS during the synthesis of UA, which poses an elevated risk to renal function (Singh et al. 2017). The data used to establish the calibration curves were listed in the Supporting Information Table S2. In our hyperuricemic mice, the oxidative stress markers, such as the levels of ROS, GSH-Px, CAT and MDA in the serum (*p* > 0.05, *F* = 0.43–1.79) and the levels of SOD, GSH-Px and CAT in the liver (*p* > 0.05, *F* = 0.34–0.56) exhibited no significant changes (Table 3). However, the levels of ROS and MDA in the liver increased significantly (*p* < 0.05, *F* = 5.68–16.42), while that of SOD in the serum decreased by 7.81% (*p* < 0.05, *F* = 6.72). Treatment with AL suppressed the levels of ROS in the serum by 9.48% and 17.86% in the liver, respectively. This treatment reduced the levels of MDA in the serum and liver by 6.66% and 11.43% (*p* < 0.05, *F* = 5.80–29.75). The administration of 10 μg BHA strongly decreased the ROS levels in both the serum and liver by 14.87% and 8.04% (*p* < 0.01, *F* = 10.50–21.99), respectively. Meanwhile, the administration of 50 µg intra-peritoneal BHA dose also significantly decreased the ROS levels in the serum and liver by 14.63% and 9.59% (*p* < 0.05, *F* = 6.64–22.43), respectively. Both 10 and 50 μg BHA treatments significantly improved liver SOD by 12.77% and 13.69% (*p* < 0.05, *F* = 8.52–16.27). Both the serum and liver levels of Cr and urea nitrogen have been accessed in this hyperuricemic mouse model (Figure 6), the levels of liver Cr increased significantly by 11.09% (*p* < 0.01, *F* = 10.95). Treatment with AL and BHA significantly lowered the levels of urea nitrogen by 25.25%, 11.65% and 12.81% (*p* < 0.05, *F* = 6.07–36.39) in the liver and the Cr by 11.52%, 8.21% and 12.02% (*p* < 0.05, *F* = 4.808–16.31) in the serum. The administration of AL and the 10 μg BHA dose dramatically lowered the levels of Cr by 15.28% and 8.14% (*p* < 0.01, *F* = 9.96–13.35), respectively in the liver.

**Table 3.** The effects of AL and BHA on the oxidative stress factors and renal function in hyperuricemic mice.

	NC	MC	AL	10BHA	50BHA
Serum					
ROS (U/mL)	252.2 ± 24.1	266.1 ± 15.8	240.9 ± 23.9*	226.6 ± 17.9***	227.2 ± 17.9***
SOD (U/mL)	171.0 ± 10.6	157.6 ± 7.3#	159.2 ± 8.0	166.6 ± 11.8	161.2 ± 8.0
GSH-PX (U/mL)	234.4 ± 10.1	238.8 ± 12.1	231.8 ± 7.7	246.8 ± 10.2	231.4 ± 13.3
CAT (U/mL)	44.4 ± 6.0	41.1 ± 4.3	41.6 ± 5.6	43.3 ± 4.6	41.5 ± 6.0
MDA (nmol/mL)	10.4 ± 0.8	10.9 ± 0.7	10.2 ± 0.5*	10.4 ± 0.5	10.5 ± 0.3
Liver					
ROS (U/mg)	67.1 ± 5.9	77.0 ± 3.6##	63.2 ± 6.2***	70.8 ± 3.7**	69.6 ± 7.1*
SOD (U/mg)	46.5 ± 2.6	50.4 ± 0.8	48.5 ± 3.4	56.8 ± 5.8*	57.2 ± 4.4**
GSH-PX (U/mg)	71.2 ± 7.1	73.9 ± 6.9	66.1 ± 7.2	78.8 ± 8.0	77.9 ± 7.3
CAT (U/mg)	13.2 ± 1.6	12.7 ± 1.6	12.9 ± 1.3	13.1 ± 1.4	14.1 ± 1.5
MDA (nmol/mg)	3.2 ± 0.3	3.5 ± 0.2#	3.1 ± 0.2**	3.4 ± 0.2	3.5 ± 0.2

Data are expressed as mean ± S.D. (*n* = 10) and analyzed via a one-way ANOVA test followed by post-hoc Dunn’s multiple comparison tests.

#*p* < 0.05 and ## *p* < 0.01 versus control mice.

*p < 0.05, **p < 0.01 and ***p < 0.001 versus model mice.

### Proteome profile on plasma of healthy volunteers and gout patients

The proteome profile in the sera of healthy volunteers and acute gout attack patients was also investigated to validate the relation between the production of cytokines and a clinical diagnosis of gout, in comparison to our animal model (Supporting Information Table S3). A cytokine array was used to detect 36 different human cytokines (Supporting Information Figure S1(A)). The results are shown on a logarithmic scale (Supporting Information Figure S1(B)). The proteome profile shows the plasma levels of interleukins (IL-1α, IL-1β, IL-1ra, IL-2, 4, 5, 6, 8, 10, IL-12 p70, IL-16, IL-17A, IL-18 and IL-27), chemokines (MCP-1, MIP-1α/β, IP-10), IFN-γ, TNF-α and TREM-1 were all increased and IL-32 was decreased in gouty patients compared to healthy volunteers (Supporting Information Table S3). However, as shown in Supporting Information Table S1 the intensities of some of those mentioned factors were very low, thus the % change may not be very convincible. Therefore, the major changes in gouty patients compared to healthy volunteers were conclude as IL-1ra, IL-8, IL-16 and IL-18 were strongly increased and IL-32 was decreased in gouty patients compared to healthy volunteers.

## Discussion

As a worldwide disease with a well-documented history, the prevalence and incidence of gout continue to increase worldwide over the past 50 years (Gabriel and Michaud 2009; Kuo et al. 2015). The increased incidence and prevalence of gout appears to be associated with the increased use of diuretics, alcohol consumption, purine intake and gastric bypass surgery, but could also be related to the lack of effective agents possessing both an anti-inflammatory and an antihyperuricemia effect (Liu et al. 2008).

Gout attacks are accompanied by the infiltration of neutrophilic granulocytes, which will produce elevated levels of ROS and lead to cell damage (Tan and Berridge 2000), as well as the release of lysosomal enzymes and inflammatory factors. Because the MSU intra-articular injection significantly increased the levels of MCP-1 as well as 6-Keto-PGF1α and depressed the level of IL-10 in the serum of rats, we believe that this rat model provides a reasonable representation of acute gout in humans. A variety of proinflammatory cytokine patterns have been reported during inflammation of joints and other tissues, are considered to play important roles in pathogenesis. For example, IL-1β is considered to play a prominent role in the cartilage breakdown in osteoarthritis (Wann et al. 2010; Sabina et al. 2011). Meanwhile, IL-8 has the effect of promoting the recruitment of inflammatory cells and increasing the production of oxidant stress mediators (Vlahopoulos et al. 1999). As was the case with the positive control drug (COL), treatment with BHA had a significant effect in alleviating ankle joint swelling induced by intra-articular MSU injection in rats. BHA treatment also significantly depressed the concentrations of IL-1β, IL-8, MCP-1 and IFN-γ, but enhanced the levels of IL-10 in the serum of rats with this model of acute gout (Table 2). As shown in Supporting Information Table S3, the levels of IL-1β, IL-8, MCP-1, IFN-γ and IL-10 were elevated significantly in the serum of patients with acute gout attack. In addition, the % change of IL-8 in gouty patients compared to healthy volunteers was 784.6%, thus the inhibition of IL-8 expression by BHA may contribute to the major anti-inflammatory role in gouty arthritis. Moreover, MCP-1, is also called CCR2, has the ability to induce chemotaxis mononuclear cells and plays an important role in rheumatoid arthritis (Xia and Sui 2009). IFN-γ promotes the development of inflammation in the lungs (Bal et al. 2016). In contrast, IL-10 is an anti-inflammatory cytokine that plays an important role in regulating immune responses and inhibiting the activation of macrophages (Greenhill et al. 2014; Ip et al. 2017). Therefore, BHA treatment showed potentials in treating gouty arthritis by acting as an anti-inflammatory agent.

HMW HA was shown to impede phagocytosis of uric acid crystals *in vitro* and the rate of the effect was proportional to the size of the HMW HA (Brandt 1974). More generally, HMW HA, through its interactions with its CD44 and RHAM receptors play a role in inflammatory responses (Misra et al. 2015). However, HMW HA play a role in experimental joint inflammation by increasing TLR2 and TLR4 expression (Campo et al. 2011), while MyD88-dependent TLR-2/TLR-4 signaling is considered to be essential for procatabolic responses to LMW HA (Liu-Bryan and Terkeltaub 2010). In addition, TLR4 signaling is also responsible for the production of chemokines (MCP-1, MIP-2) and cytokines (TNF-α, IL-6) that are involved in the inflammation (Pushpakumar et al. 2017). The development of TLR4 inhibitors and the strategies to block TLR4 signaling has demonstrated the potential to treat inflammation and oxidative stress related diseases (Carrillo-Sepulveda et al. 2015; Hsu et al. 2017), indicating that there is a close relationship between TLR4 signaling and oxidative stress. We have previously shown that the anti-inflammatory activity of BHA against LPS was appeared to be mediated through the interaction with the TLR4 receptor on the surface of the cells (Babasola et al. 2014). Thus, the anti-acute gout activity of BHA could be achieved through interaction with the cell surface receptor TLR4. In the meantime, UA crystals can also interact with the TLR4 receptor on the surfaces of cells to activate the TLR4-NLRP3 inflammasome, leading to caspase-1-dependent cleavage of pro-IL-1β, thereby triggering the release of IL-1β (Liu et al. 2017). Therefore, the mechanism of the anti-inflammatory effects of BHA could be through the binding of BHA to TLR4 to prevent TLR4 from interacting with UA, thus regulating the NF-κB signaling pathway (Babasola et al. 2014; Desai et al. 2017; Liu et al. 2017). In addition, the elevated concentration of IL-10 in the serum of gouty patients (see the Supporting Information file) could be related to the feedback regulation.

Hyperuricemia is considered as a necessary condition for the development of gout, and XO that plays a key catalytic role in UA production (Meneshian and Bulkley 2002). Our results indicated that the levels of serum UA and liver XO increased significantly in hyperuricemia mice and oral administration with AL resulted in reduction of the serum UA to normal levels and this was also the case with BHA, which significantly reduced the serum UA levels (Figure 4(A)). The dose of 10 μg BHA intra-peritoneally dramatically reduced liver XO activity. Thus, the antihyperuricemic activity of BHA was achieved partly by inhibiting the activity of liver XO and thus reducing the serum UA levels (Ma et al. 2018). However, treatment with 50 μg BHA had no significant effect on XO activity in the livers of hyperuricemic mice but decreased the serum UA levels as the case of AL (Figure 4(B)). It was not surprising to see that although AL decreased uric acid level in serum *in vivo*, the activity of XO in liver homogenate was not affected *in vitro*. Because AL acts as an inhibitor of XO, which not affects the expression level of XO, this result could be due to the fact that in the *in vitro* assays, the activity of XO in liver homogenate was determined by adding exogenous XO substrates, but without the addition of AL. In the meantime, 10 μg BHA showed a better XO inhibitory effect *in vitro* and exhibited similar serum uric acid-lowering effects with 50 μg BHA as well as with AL. This result suggests that BHA may lower uric acid with a different mechanism, such as down-regulate the expression of XO in liver, and lower dosage exhibited a better effect. However, its mechanisms of action of uric acid-lowering remain unrevealed.

XO would catalyze the oxidation of hypoxanthine to xanthine and xanthine to uric acid, which would be accompanied by a large amount of ROS (Singh et al. 2017). In hyperuricemic mice the levels of ROS and MDA were significantly elevated in the liver. MDA is formed via the degradation of polyunsaturated fat by ROS, and thus could be considered as a biomarker for oxidative damage (Davey et al. 2005). The content of superoxide dismutase (SOD, an important enzyme in the control of ROS, controls the oxidative stress) in the serum was significantly diminished. It has been reported that TLR4 is involved in ROS generation during an inflammation reaction and the attenuation of ROS production can be achieved via down regulation of TLR4 (Sahnoun et al. 2017; Jiang et al. 2018), and TLR4 deficiency inhibits ROS production and increases the antioxidant activities of superoxide dismutase and catalase, thus alleviating inflammation reaction (Carrillo-Sepulveda et al. 2015; Pushpakumar et al. 2017). BHA treatment significantly decreased the levels of ROS in the serum and liver and increased the level of SOD in the livers of hyperuricemia mice, which in consistent with the observed lowered serum UA levels. These effects could be achieved through the interaction of BHA with TLR4.

Hyperuricemia has been closely associated with renal dysfunction and the side-effects of drugs such as NSAIDs also can cause acute renal failure as indicated by elevated serum Cr and blood urea nitrogen levels (Singh et al. 2003; Levey et al. 2006; Vaidya et al. 2006). In our study, we have measured both the Cr and urea nitrogen in serum and in liver to access the pathogenesis change in this hyperuricemia mouse model as well as to evaluate the safety of BHA treatment (Figure 6). In our hyperuricemic mice the level of liver Cr increased significantly while there was no substantial increase of serum Cr. As was the case with treatment of the hyperuricemia mice with AL, treatment with BHA significantly reduced the urea nitrogen in liver and Cr in the serum and liver. As discussed above, BHA treatment significantly reduces the level of ROS in hyperuricemic mice and thus could provide a protective effect on renal function as AL (Krishnamurthy et al. 2017).

Commercial preparations of HMW HA administered intra-articularly have been studies mostly in the treatment of osteoarthritis of weight of bearing joints and may provide some benefit similar to glucocorticoids (Bannuru et al. 2009) and NSAIDs (Bannuru et al. 2014). In the treatment of gout both NSAIDs and intra-articular corticosteroids are used clinically, but intra-articular HA does not have significant usage (Ragab et al. 2017). Further, there are no reports regarding an antihyperuricemic effect of HA. In this study, we have synthesized a LMW HA derivative, BHA, and evaluated its effects and mechanisms of action in animal models of acute gouty arthritis and in rats and in hyperuricemic mice. Our results suggest that BHA may have translational potential in the treatment of acute and chronic gout.

## Conclusions

BHA inhibited the development of ankle swelling due to the inflammatory reaction induced by intra-articular MSU injection in rats. Its intra-peritoneal administration also reduced the serum UA concentration in mice with hyperuricemia, which was induced by OXO and yeast extract powder. These preventive anti-acute gout and antihyperuricemia effects of BHA were associated with the inhibition of inflammatory factors (IL-1β, IL-8, MCP-1 and IFN-γ) and the promotion of the anti-inflammatory cytokine (IL-10), which in turn reduces the level of inflammation and inhibits the XO activity by regulating oxidative stress. At the same time, BHA also protects the renal function of the hyperuricemic mice. In conclusion, BHA is a novel compound, derived from a naturally occurring carbohydrate polymer, which has translational potential for the clinical treatment of gout due to its bimodal pharmacological activity.

## Supplementary Material

BHA-Supplement_data-20190903.doc
