# Supplementary material for: N-Butyrylated hyaluronic acid ameliorates gout and hyperuricemia in animal models
Source: Pharm Biol. 2019 Oct 17;57(1):717–28. doi: 10.1080/13880209.2019.1672755 (PMC8871623; doi:10.1080/13880209.2019.1672755)
Supplement: BHA-Supplement_data-20190903.doc [file IPHB_A_1672755_SM8324.doc]

**Supplementary data**

**Table S1.** Absorbance recorded in different concentrations that used to establish the calibration curves to determine the levels of cytokines with the corresponding ELISA kit. The calibration curve with R^2^ > 0.99 is considered accurate.

| **Cytokines** | **Absorbance recorded in different concentrations (pg/mL)** | | | | | | | **R^2^** |
| --- | --- | --- | --- | --- | --- | --- | --- | --- |
| IL-1α | Concentration | 0 | 3 | 6 | 12 | 24 | 48 | 0.9953 |
|  | Absorbance | 0.092 | 0.074 | 0.214 | 0.339 | 0.64 | 1.253 |  |
| IL-1β | Concentration | 0 | 2.5 | 5 | 10 | 20 | 40 | 0.9996 |
|  | Absorbance | 0.046 | 0.157 | 0.25 | 0.461 | 0.823 | 1.574 |  |
| IL-6 | Concentration | 0 | 10 | 20 | 40 | 80 | 160 | 0.9999 |
|  | Absorbance | 0.054 | 0.118 | 0.193 | 0.326 | 0.626 | 1.195 |  |
| IL-8 | Concentration | 0 | 15 | 30 | 60 | 120 | 240 | 0.9986 |
|  | Absorbance | 0.076 | 0.125 | 0.183 | 0.351 | 0.661 | 1.208 |  |
| IL-10 | Concentration | 0 | 1.5 | 3 | 6 | 12 | 24 | 0.9978 |
|  | Absorbance | 0.05 | 0.139 | 0.234 | 0.424 | 0.71 | 1.487 |  |
| IL-16 | Concentration | 0 | 25 | 50 | 100 | 200 | 400 | 0.9985 |
|  | Absorbance | 0.052 | 0.13 | 0.219 | 0.356 | 0.679 | 1.397 |  |
| IP-10 | Concentration | 0 | 10 | 20 | 40 | 80 | 160 | 0.9980 |
|  | Absorbance | 0.077 | 0.136 | 0.21 | 0.355 | 0.619 | 1.27 |  |
| MCP-1 | Concentration | 0 | 50 | 100 | 200 | 400 | 800 | 0.9920 |
|  | Absorbance | 0.058 | 0.135 | 0.19 | 0.389 | 0.686 | 1.146 |  |
| MIP-1α | Concentration | 0 | 20 | 40 | 80 | 160 | 320 | 0.9936 |
|  | Absorbance | 0.047 | 0.097 | 0.171 | 0.305 | 0.671 | 1.142 |  |
| IFN-γ | Concentration | 0 | 7.5 | 15 | 30 | 60 | 120 | 0.9994 |
|  | Absorbance | 0.051 | 0.129 | 0.194 | 0.337 | 0.539 | 1.313 |  |
| TNF-α | Concentration | 0 | 20 | 40 | 80 | 160 | 320 | 0.9993 |
|  | Absorbance | 0.047 | 0.131 | 0.215 | 0.401 | 0.699 | 1.329 |  |
| TNF-β | Concentration | 0 | 15 | 30 | 60 | 120 | 240 | 0.9952 |
|  | Absorbance | 0.058 | 0.186 | 0.304 | 0.496 | 0.798 | 1.443 |  |
| NF-κB | Concentration | 0 | 75 | 150 | 300 | 600 | 1200 | 0.9983 |
|  | Absorbance | 0.119 | 0.138 | 0.183 | 0.246 | 0.359 | 0.635 |  |
| 6-Keto-PGF1α | Concentration | 0 | 50 | 100 | 200 | 400 | 800 | 0.9955 |
|  | Absorbance | 0.047 | 0.099 | 0.165 | 0.291 | 0.507 | 0.872 |  |
| PGE2 | Concentration | 0 | 30 | 60 | 120 | 240 | 480 | 0.9990 |
|  | Absorbance | 0.05 | 0.149 | 0.259 | 0.446 | 0.824 | 1.514 |  |

**Table S2.** Absorbance recorded in different concentrations that used to establish the calibration curves to determine the levels of the oxidative stress factors and renal function related factors with the corresponding ELISA kit.

| **Factors** | **Absorbance recorded in different concentrations/activities** | | | | | | | **R^2^** |
| --- | --- | --- | --- | --- | --- | --- | --- | --- |
| ROS | Activities (U/mL) | 0 | 30 | 60 | 120 | 240 | 480 | 0.9985 |
|  | Absorbance | 0.046 | 0.111 | 0.167 | 0.317 | 0.576 | 1.193 |  |
| SOD | Activities (U/mL) | 0 | 20 | 40 | 80 | 160 | 320 | 0.9998 |
|  | Absorbance | 0.046 | 0.104 | 0.164 | 0.308 | 0.567 | 1.101 |  |
| GSH-Px | Activities (U/mL) | 0 | 30 | 60 | 120 | 240 | 480 | 0.9987 |
|  | Absorbance | 0.058 | 0.148 | 0.239 | 0.454 | 0.758 | 1.528 |  |
| CAT | Activities (U/mL) | 0 | 5 | 10 | 20 | 40 | 80 | 0.9991 |
|  | Absorbance | 0.047 | 0.102 | 0.175 | 0.348 | 0.708 | 1.395 |  |
| MDA | Concentration (nmol/mL) | 0 | 1.5 | 3 | 6 | 12 | 24 | 0.9991 |
|  | Absorbance | 0.047 | 0.152 | 0.205 | 0.433 | 0.762 | 1.485 |  |
| Urea nitrogen | Concentration (mmol/L) | 0 | 1.5 | 3 | 6 | 12 | 24 | 0.9948 |
|  | Absorbance | 0.045 | 0.139 | 0.213 | 0.425 | 0.78 | 1.343 |  |
| Creatinine | Concentration (μmol/L) | 0 | 15 | 30 | 60 | 120 | 240 | 0.9991 |
|  | Absorbance | 0.062 | 0.151 | 0.226 | - | 0.893 | 1.675 |  |

**Table S3. The Instruction of Protein Chip ary005b**

|  | Name | Healthy | Gouty | change rate |
| --- | --- | --- | --- | --- |
| A3，A4 | CCL1/I-309 | 0.0301 ± 0.0018 | 0.0320 ± 0.0072 | 6.0% |
| **A5，A6** | **CCL2/MCP-1** | **0.0282 ± 0.0076** | **0.0363 ± 0.0052** | **28.6%** |
| **A7，A8** | **MIP-1α/MIP-1β** | **0.0260 ± 0.0034** | **0.0551 ± 0.0244** | **111.7%** |
| A9，A10 | CCL5/RANTES | 1.0209 ± 0.0163 | 0.9880 ± 0.0512 | -3.2% |
| A11，A12 | CD40 Ligand/TNFSF5 | 0.6125 ± 0.1103 | 0.7003 ± 0.0124 | 14.3% |
| A13，A14 | Complement Component C5/C5a | 0.8695 ± 0.0340 | 0.9158 ± 0.0406 | 5.3% |
| A15，A16 | CXCL1/GROα | 0.2360 ± 0.1070 | 0.2440 ± 0.1417 | 3.4% |
| **A17，A18** | **CXCL10/IP-10** | **0.0414 ± 0.0033** | **0.0562 ± 0.0159** | **35.6%** |
| B3,B4 | CXCL11/I-TAC | 0.0690 ± 0.0206 | 0.0574 ± 0.0230 | -16.9% |
| B5,B6 | CXCL12/SDF-1 | 0.8340 ± 0.1538 | 0.8016 ± 0.1069 | -3.9% |
| B7,B8 | G-CSF | 0.0408 ± 0.0029 | 0.0435 ± 0.0050 | 6.6% |
| B9.B10 | GM-CSF | 0.0418 ± 0.0061 | 0.0475 ± 0.0064 | 13.5% |
| B11,B12 | ICAM-1/CD54 | 0.9964 ± 0.0131 | 1.1004 ± 0.0765 | 10.4% |
| **B13,B14** | **IFN-γ** | **0.0402 ± 0.0087** | **0.0656 ± 0.0080** | **63.1%** |
| **B15,B16** | **IL-1α/IL-1F1** | **0.0420 ± 0.0013** | **0.0657 ± 0.0102** | **56.5%** |
| **B17,B18** | **IL-1β/IL-1F2** | **0.0317 ± 0.0046** | **0.0549 ± 0.0042** | **73.1%** |
| **C3,C4** | **IL-1ra/IL-1F3** | **0.2637 ± 0.0475** | **0.5367 ± 0.0530** | **103.5%** |
| **C5,C6** | **IL-2** | **0.0409 ± 0.0042** | **0.0581 ± 0.0103** | **42.0%** |
| **C7,C8** | **IL-4** | **0.0306 ± 0.0066** | **0.0529 ± 0.0163** | **73.2%** |
| **C9,C10** | **IL-5** | **0.0359 ± 0.0098** | **0.0537 ± 0.0117** | **49.5%** |
| **C11,C12** | **IL-6** | **0.0567 ± 0.0154** | **0.0785 ± 0.0050** | **38.6%** |
| **C13,C14** | **IL-8** | **0.0368 ± 0.005** | **0.3258 ± 0.3110** | **784.6%** |
| **C15,C16** | **IL-10** | **0.0358 ± 0.0066** | **0.0587 ± 0.0039** | **63.8%** |
| **C17,C18** | **IL-12 p70** | **0.0365 ± 0.0012** | **0.0606 ± 0.0023** | **66.1%** |
| D3,D4 | IL-13 | 0.1187 ± 0.0453 | 0.1342 ± 0.0345 | 13.0% |
| **D5,D6** | **IL-16** | **0.1793 ± 0.0302** | **0.3626 ± 0.1027** | **102.2%** |
| **D7,D8** | **IL-17A** | **0.0307 ± 0.0087** | **0.0532 ± 0.0168** | **73.4%** |
| D9,D10 | IL-17E | 0.0634 ± 0.0219 | 0.0686 ± 0.0412 | 8.2% |
| **D11,D12** | **IL-18/IL-1F4** | **0.1149 ± 0.0235** | **0.2276 ± 0.0328** | **98.0%** |
| D13,D14 | IL-21 | 0.0798 ± 0.0344 | 0.0931 ± 0.0415 | 16.7% |
| **D15,D16** | **IL-27** | **0.0382 ± 0.0044** | **0.0494 ± 0.0107** | **29.4%** |
| **D17,D18** | **IL-32a** | **0.1314 ± 0.0921** | **0.0920 ± 0.0555** | **-30.0%** |
| E3,E4 | MIF | 0.7833 ± 0.1083 | 0.8127 ± 0.0328 | 3.8% |
| E5,E6 | Serpin E1/PAI-1 | 0.9780 ± 0.0297 | 1.0493 ± 0.0558 | 7.3% |
| **E7,E8** | **TNF-α** | **0.0307 ± 0.0109** | **0.0561 ± 0.0203** | **82.4%** |
| **E9,E10** | **TREM-1** | **0.0260 ± 0.0157** | **0.0499 ± 0.0203** | **92.2%** |

Blood was sampled from 6 health volunteers and 6 patients suffered with acute gout attack, who have been enrolled in the first hospital of Jilin University, Changchun, China. Inflammatory factors of human plasma were assayed using a Proteome Profiler Human Cytokine Array Kit (Ary005b, R&D Systems Bio-Technology China Co. Ltd, Minneapolis, USA). Change rate = (gouty – healthy)/healthy. Data are expressed as mean ± S.D. (n = 6) and analyzed by using one-way ANOVA followed by post-hoc Dunn’s multiple comparisons test. **Bold**, changes > 20%.


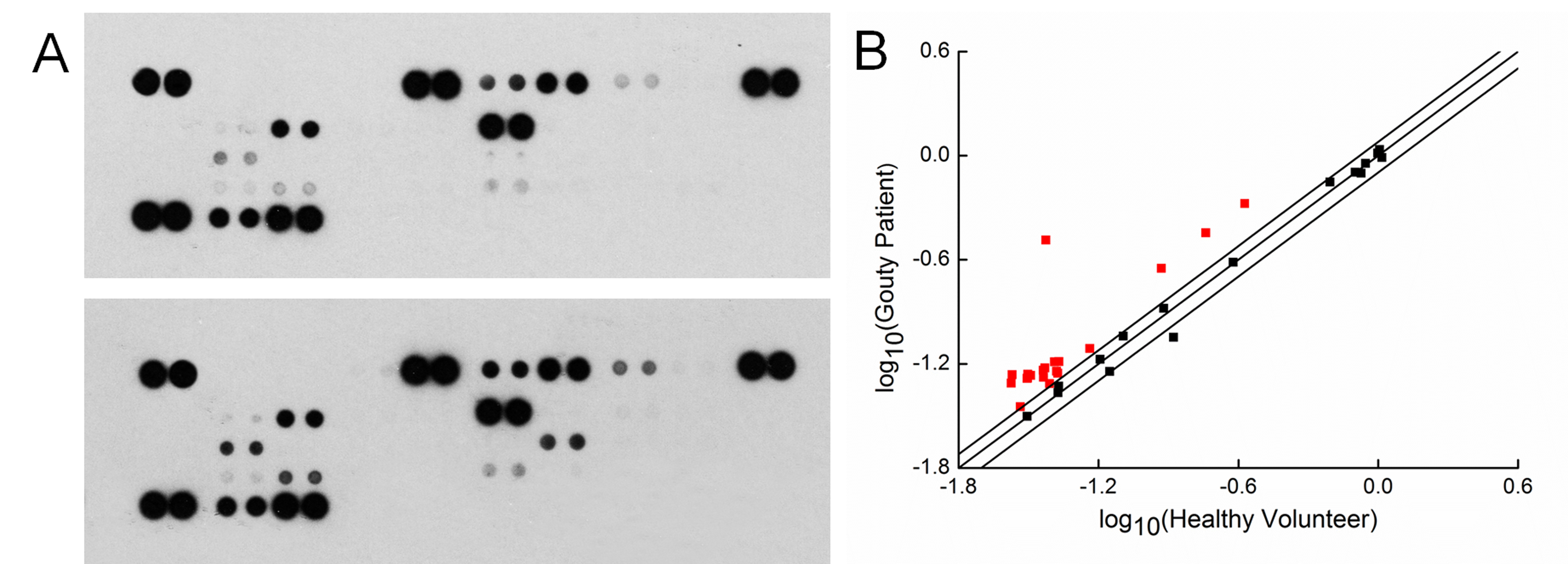


**Figure S1. (A)** The picture of the Proteome Profiler Human Cytokine Array Kit detected 36 different human cytokines of healthy volunteers and gouty patients (n = 6). **(B)** The logarithm results of cytokine array, and ± 20% changes limit lines were provided in the picture.
